# Analysis of the Impact of Changes in Thermomechanical Properties of Polymer Materials on the Machining Process of Gears

**DOI:** 10.3390/polym13010028

**Published:** 2020-12-23

**Authors:** Adam Gnatowski, Rafał Gołębski, Piotr Sikora

**Affiliations:** Department of Technology and Automation, Czestochowa University of Technology, Armii Krajowej Ave. 21, 42-200 Czestochowa, Poland; piotr@apjsikora.pl

**Keywords:** thermomechanical properties, melting, glass transition, annealed, CNC machining, polymeric materials roughness, spur gear, step by step method

## Abstract

This paper presents an analysis of the impact of modification of thermomechanical properties of polymer materials on the process of gear wheel machining on a CNC machine tool. Polymer materials Tecaflon (PVDA) and polyethylene (PE) were used for processing. The materials underwent thermal modification i.e., annealing. Prepared samples (gear wheel dimensions Ø76.5 × 20 mm) were machined under the same conditions, only changing the feed rate parameter. A CNC milling machine of its own construction was used for machining with a horizontal numerical dividing attachment. The obtained gear wheels were tested using ZEISS GEAR PRO gear analyzes software. Deviations of the involute outline and the tooth line allowed classification of wheels in the 9th grade of accuracy. Machined teeth surfaces were examined for changes in the properties of surface layer, taking into account the influence of polymer material thermal modification on the surface condition. The samples were tested for mechanical properties (tensile strength) and thermomechanical properties (DSC and DMTA). The tests showed positive changes in material strength and significant improvements in PVDA Tecaflon after heat treatment.

## 1. Introduction

Involute cylindrical gears with straight teeth are most commonly used in drive units in many machines and devices. They are most often machined using shaping, presented by Litvin and Fuentes (2004) [1], or with the envelope method presented by Boral et al. (2018) [2]. In the case of series machining, for example, bevel gears for differential gears, new technologies are used, such as envelope burnishing and accurate forging, with these this two types of machining described by Furst (2012) [3]. Gears made of polymer materials are increasingly being used in the machine industry as described by Drummer et al. (2012) [4], and they are beginning to be commonly used due to their acid resistance and lower noise emission, which was the topic investigated by Uysal et al. (2011) [5]. These polymers are characterized by many advantages, which include: chemical resistance, the ability to dampen vibrations and as a result, low noise emission, low coefficient of friction, high abrasion resistance, self-lubricity, good insulating properties, low weight and aesthetic appearance, as presented by Bussey et al. (2018) [6]. In the case of machining, gears are usually processed with the use of special tools on machines designed for this specific type of machining. Golebski (2016) [7] described that the constant development of modern machine tools and cutting tools contributes to gear construction correction and improvement of gears machining technology, which he also presented in his work with Andrei et al. (2005) [8]. The most efficient and the most accurate method of machining cylindrical gears is the envelope machining using modular hobs cutters. This technology has been widely used for a very long time. The surfaces of the hob and the machined wheel create a technological gear type, i.e., worm gear–worm wheel, in the machining process. The surface of the hob and the machined wheel are reciprocally enveloped, so the hob geometry is strictly defined. Modular hobs have a very complex geometry and are technologically difficult to manufacture. In addition, modular hobs with high accuracy are very expensive. In the case of tooth profile modification, special tools with a modified outline need to be used, which entails generation of costs and extends the time of production preparation.

This article presents the technology of machining cylindrical gears with straight teeth using the step by step method (sbs) on a CNC machine tool. The polymer materials PVDA Tecaflon and polyethylene (PE) were used for processing. In order to predict the changes in material properties during the machining process and during the exploitation of gears, we tested the thermal dynamic properties by DMTA, as well as thermal properties by differential scanning calorimetry (DSC), and tensile strength tests were also carried out. The tests were carried out for polymers before and after heat treatment, aimed at improving the material treatment process and the properties of manufactured gears. The tool used for machining was a ball end mill, whose geometry was not related to the geometry of the machined teeth; the characteristics of the tool are described in detail in the Perschmann (2018) catalogue [9]. The developed technology allowed machining of gears with longitudinal and transverse modification of the tooth profile, as was presented by Golebski (2016) [7]. It can be especially useful when machining wheels in unit production, wheels with large modules and with modification of teeth. This paper presents the case of machining gears made of polymer materials. However, the considerations are universal, and the machining method is compatible with the latest global trends in this field, and is increasingly used to treat other gears and worm gears described in work by Nieszporek et al. (2017) [10].

## 2. Methodology of Conducted Tests and Determination of Processing Parameters

Tecaflon (PVDA) and ultra-high molecular polyethylene PE1000 manufactured by ENSINGER were used for the tests. The polymers selected for testing should have good machinability. There is not much information in the literature on the selection of tool geometry for machining polymer materials. Moreover, there are a lot of polymer materials and new ones are still being produced. During polymer processing, problems occur related to plasticity such as a low Young’s modulus and low thermal conductivity of these materials. Too much heat and surface pressures during processing can cause distortion and, consequently, deterioration of the machined surface and assumed dimensional tolerances, as was presented by Rudy (2004) [11]. Samples complying with the PN-EN ISO 6892-1: 2010 standard were used for comparative tests. Samples for testing were warmed up at 80 °C for PE1000 and at 120 °C for PVDA Tecaflon. The samples were soaked for 900 s and the heating velocity was 0.015 °C/s while the cooling took place at a rate of 0.01 °C/s. This paper presents the results of investigations of thermal properties performed with the differential scanning calorimetry method using a NETZSCH PC 200 device for polymeric materials before and after heat treatment. Samples for DSC testing were cut from the core of a Ø80 mm shaft used for processing and were weighed with SARTORIUS balances with 0.01 mg precision, using the internal calibration option and a closed weighing space. The weight of the test samples ranged from 7 to 12 mg. DSC curves were recorded when heating samples and cooling them at a rate of 10 °C/min in the temperature range given in Table 1. The thermal parameters of the test samples were analyzed using the NETZSCH software. This software allows us to determine the thermal parameters of the sample in a given temperature range and to determine the area between the thermographic curve and the baseline in the scope of the endothermic effect. The analysis of dynamic thermal properties was carried out with the NETZSCH DMA 242 device with a holder for three-point free bending of the sample in the form of a beam with dimensions of 50 × 10 × 4 mm^3^. The samples were fixed in the holder and were subjected to a sinusoidal force of 1 Hz and 10 Hz with a constant amplitude, at a heating rate of 3 °C/min to the temperature selected on the basis of DSC results (Table 2). The values of the elastic modulus E’ and the mechanical loss factor tan δ were calculated on the basis of the values of forces and strain in relation to the dimensions of the samples; the interpretation of results and the preparation of samples are explained in [12]. Tensile strength tests were carried out according to the standard PN-EN ISO 527-2: 2012. The samples were tested on an electromechanical tensile testing machine type ZWICK100 with a measuring range of 0–100 kN. Table 1 presents the scope of measurement of research samples by DSC and DMTA.

**Figure 1:**
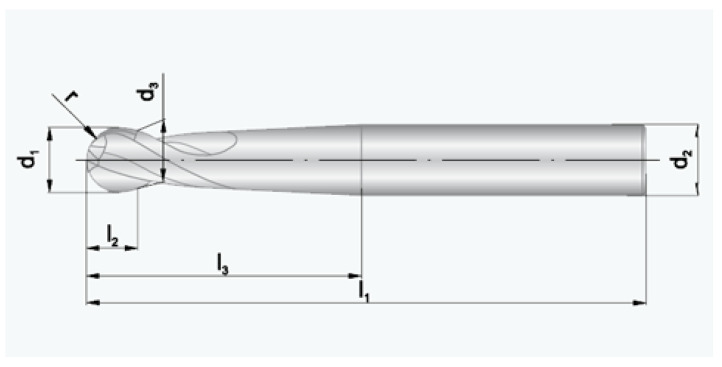
Ball end milling cutter used in processing (Perschmann 2020).

In the processing of polymer materials, large rotational speeds of the tool are recommended for medium feeds and a small angle of rake, which allows us to obtain a good surface quality. The milling cutters for polymer processing should be characterized in the geometry with the largest possible chip channels, in order to facilitate the removal of the chip along with the heat generated during processing. For this reason, a maximum of two blade tools are recommended. It is also recommended to use diamond coatings to facilitate chip removal, however, such tools are several times more expensive than tools with standard coatings. The use of a tool with a small number of blades also reduces vibrations of the tool, which affects the quality of the machined part. For polymer milling, cutters with geometry of the clearance angle *α* = 5–0° and rake angle ℽ = 0–12° are recommended. Generally, mill cutters for polymer machining should have fewer cutting edges than regular tools for steel machining; furthermore, they should be characterized by high clearance and rake angles. A tool with a geometry dedicated to soft and ductile materials was used for machining of the tested materials, with such an application described in the catalogue by Perschmann (2018) [9]. This is a 5 mm ball end solid carbide cutter with polished chip flutes (Figure 1), used as an alternative to an expensive diamond-coated tool. The following cutting parameters were used: feed per tooth fz = 0.05–0.07 mm/blade, cutting speed v = 200–500 m/min. In order to obtain a high machining efficiency, and also due to the step by step machining with use of a small diameter cutter, cutting parameters much higher than those generally accepted for steel processing were used. A cylindrical gear wheel was designed with the following parameters: module m = 4.5 mm, longitudinal modification of the tooth e = 0.16 mm, width of the wheel b = 20 mm, number of teeth = 15. The number of tool passes at the height of the outline was assumed at 115.

**Figure 2:**
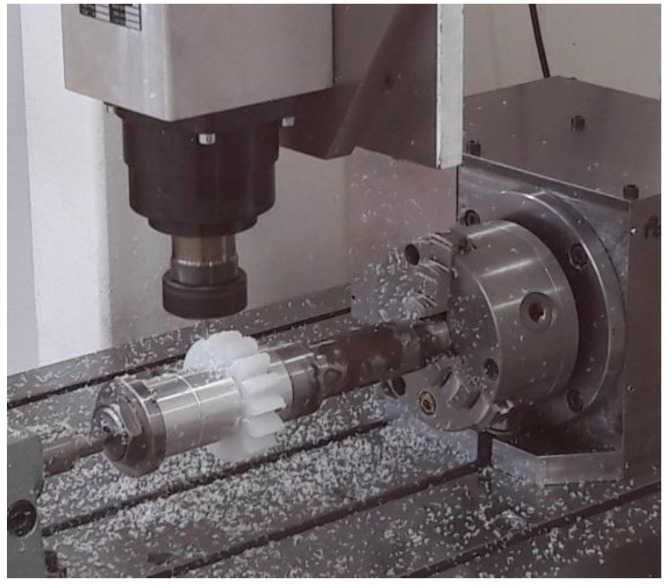
Machining the gear, material PE 1000 after heat treatment.

The gears were cut on a three-axis milling machine of their own design with a numerically controlled horizontal dividing attachment described by Boral et al. (2017) [13]. The entire machine tool construction was based on closed steel profiles with a large cross-section and a high wall thickness. This allowed us to obtain a rigid body structure. To obtain high accuracy of displacements, high accuracy profiled guideways were used, on which linear ball bearings with pre-tension moved. The main drive of the milling machine was a 5.5 kW electro-spindle with a maximum rotational speed of 18,000 rpm with an ER32 end for clamping tools with a cylindrical shank. The workspace in each axis x/y/z was 450/720/320 mm, respectively. The used machine tool is mainly designed for machining with small diameter mill cutters using the step by step method on geometrically complex surfaces. The fourth axis allows for milling of rotating surfaces and teeth (Figure 2).

**Figure 3:**
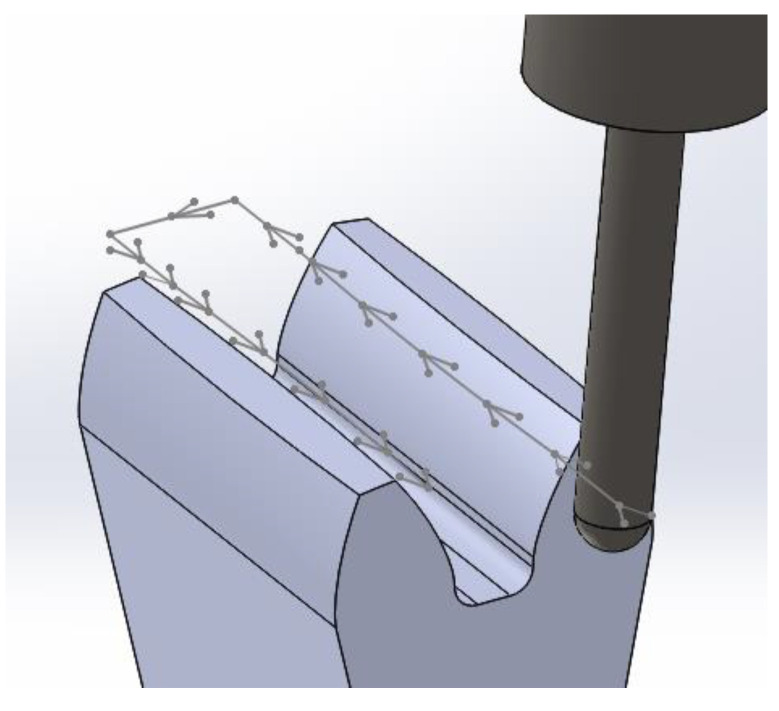
Strategy for machining the gear tooth.

Four previously prepared samples were machined in the following order: PVDA, PVDA after heat modification, PE1000 and PE1000 after thermal modification. The developed machining program included roughing in the strategy of removing as much material as possible in one tool pass, and finishing in the strategy of equal machining allowance at the height of the tooth at each tool transition. During the machining, oil mist lubrication was used, feeding the lubricant to the machined surface every 45 s for 4 s. In addition, during the continuous machining, the chips were removed using compressed air. Throughout the entire machining process, the conditions were identical for all 4 machined gears. The tool speed was assumed to be 16,000 rpm. The feed rates used were, respectively: 2000, 2600, 3200, 4000 (mm/min). The gear was machined with increasing feed rates in 4 zones around the perimeter of the gear, which resulted in the processing of 3 sections between tooth with each feed. An up milling type was used for all machining cases. The tool with the same diameter was used to machining the flank and face of the tooth, fillet radius and outline modification (Figure 3).

## 3. The Results and Discussion

**Figure 4:**
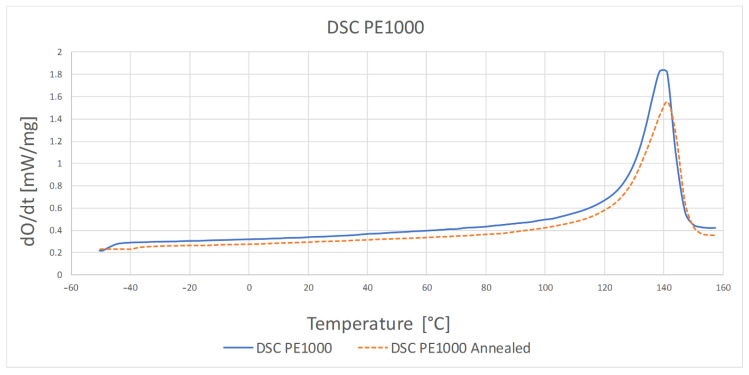
DSC thermogram for PE1000 before and after heat treatment.

**Figure 5:**
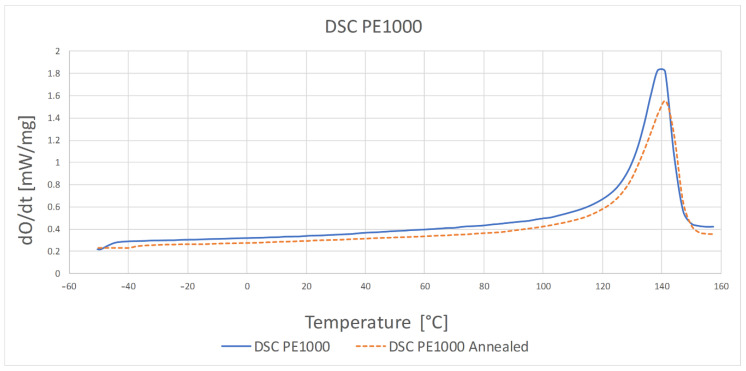
DSC thermogram for PVDA Tecaflon before and after heat treatment.

The results of the differential scanning calorimetry test are presented in Table 2 and Figure 4 and Figure 5.

**Figure 6:**
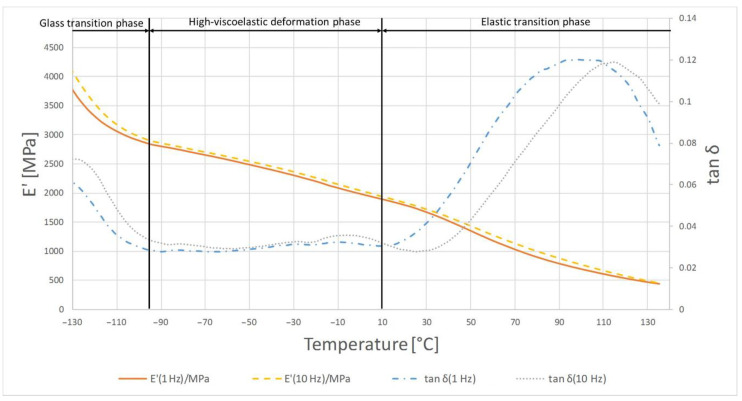
Results of DMTA tests for PE1000.

**Figure 7:**
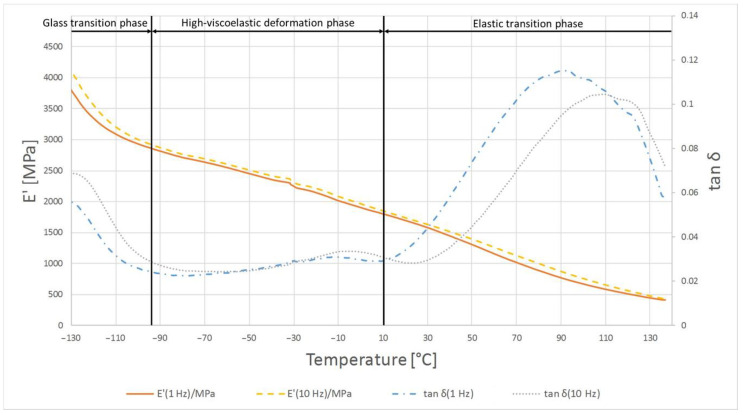
Results of DMTA test for PE1000 after heat treatment.

**Figure 8:**
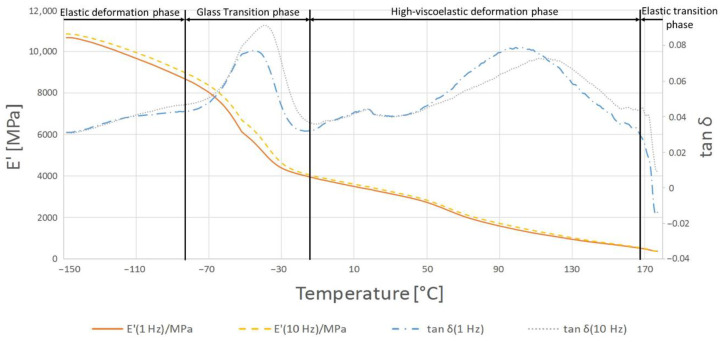
Results of DMTA test for PVDA Tecaflon.

**Figure 9:**
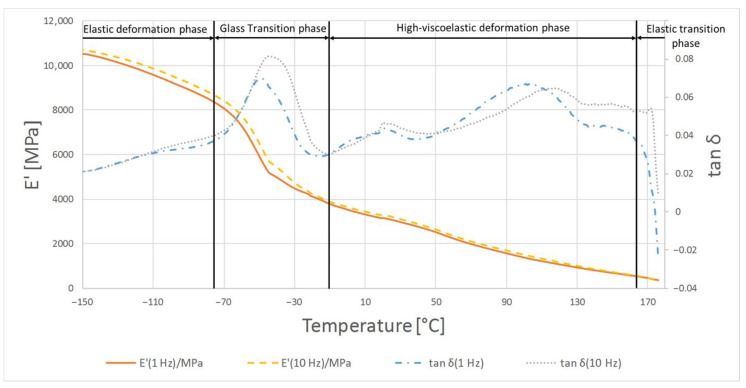
The results of DMTA test for PVDA Tecaflon after heat treatment.

The highest temperature at which the crystalline phase melted was PE1000 at 175.7 °C and the lowest melting point was characterized by PVDA Tecaflon at 139.8 °C. The crystalline melting range was the widest for PVDA Tecaflon after heat treatment at 24.8 °C, the narrowest was for PE1000 at 10.4 °C. The highest melting enthalpy value was recorded for PVDA Tecaflon at 146.4 J/g and the lowest for PE1000 at 55.87 J/g. In the case of PVDA Tecaflon, an increase in melting enthalpies by 13.22% was recorded for the annealed samples, while an increase in melting enthalpies was not recorded for PE. Wootthikanokkhan et al. (2013) [14] presented results from DSC before and after annealing. The increase in the melting enthalpy value and, consequently, the degree of crystallinity of the polymer after annealing, affects the hardness and stiffness of the material, which is important in the machining process, chip formation and surface quality of the obtained product. The results of the analysis of dynamic mechanical properties are shown in Figure 6, Figure 7, Figure 8 and Figure 9. The graphs show changes in the modulus of elasticity and tangent of the angle of mechanical losses depending on the temperature at 1 and 10 Hz.

Figure 6 shows the temperature dependence of the tan δ mechanical loss angle and the retention module E’ for PE1000. At the beginning of the glass transition phase, a very large dependence of the loss modulus E’ from the temperature is noticeable, while at higher temperatures its dependence decreases, and from −90 °C it changes to a constant temperature dependence. In this phase tan δ first shows a high temperature dependence and there is a relaxation conversion in the polymer. The increase of the tan δ value is then registered from −20 °C, at 30 °C there is a very large temperature dependence, and at 100 °C there is a maximum 0.12 at 1 Hz frequency. Above 110 °C in the material flow phase, the E’ value is constantly decreasing, showing a moderate temperature dependence.

In the case of material samples after heating, the first phase shows differences in relation to PE1000 not annealed where tan δ begins to decrease from smaller values. The maximum tan δ value was 0.115 at 1 Hz. In the material flow phase, temperature compliance E’ is similar for samples before and after heating, while tan δ values are lower.

The glass transition phase was recorded from the beginning of the temperature range tested up to −45 °C where tan δ reaches its maximum of 0.07; in this temperature range the storage module shows a very strong temperature dependence and decreases from 10,800 to 6000 MPa. In the phase of highly elastic deformations, tan δ decreases, showing a high temperature dependence of up to 0.03, then it increases to 0.079 at 100 °C at 1 Hz. In this respect, the E’ module exhibits a stronger temperature dependence than in the previous phase and decreases from 6000 to 1440 MPa. In the flow phase of the material under test, the E’ and tan δ modules are characterized by a slow drop in value when the temperature rises, reflecting similar research by Gnatowski (2010) [15].

**Figure 10:**
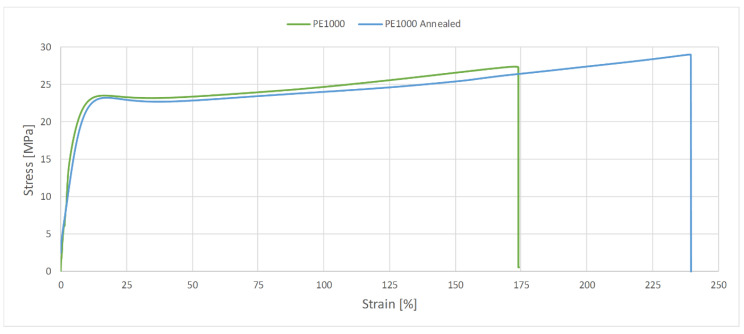
Diagram of the relationship between tensile strength and elongation for PE1000 and PE1000 after heat treatment.

**Figure 11:**
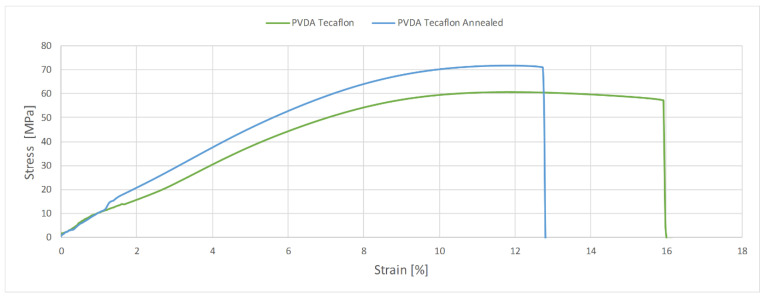
Diagram of the relationship between tensile strength and elongation for PVDA Tecaflon and PVDA Tecaflon after heat treatment.

The glass transition phase for the annealed PVDA Tecaflon is very similar to how it works in the case of thermogravimetric curve analysis for PVDA Tecaflon, with the difference being that the relaxation transition and visible maximum tan δ reaches 0.068 and E’ stops at 5700 MPa. In the next phase, analogous changes of tan δ were also recorded, the values of which initially decreased, then gradually reached a maximum of 0.06 at 100 °C. Analyzing the results of the research, it can be noticed that Tecaflon became stiffer when heated and the dependence of E’ on the temperature also increased. The flow phase of the tested material is slightly different, because after a strong influence of temperature on tan δ there is a slight effect of temperature on tan δ, whose values gradually decrease. In the case of all tested samples of different materials, changes in the values of tangent loss curves were recorded at different frequencies 1 and 10 Hz both before and after heating, however the characterization of the curves for variable frequencies is the same. Methods and procedures of DMTA tests are described in [16]. Figure 10 and Figure 11 show graphs for samples subjected to the tensile strength test, while in Table 3 the maximum forces at which the polymers were broken off are summarized.

In the case of PVDA Tecaflon, annealing resulted in increased strength, a higher value of the force required to break the sample was recorded and the force increased from 2426 N and strength 60.67 MPa to 2874 N and 71.86 MPa. A decrease in elongation during the PVDA Tecaflon tensile test after annealing by 4% was also recorded. The EP1000 annealed is characterized by a lower value of force required to break the sample: PE1000 breaks at 1069.1 N and the strength is 27.40 MPa. In contrast, heat-treated Tecaflon ruptures at 1160.6 N and strength is 29.02 MPa. In the case of PE1000, the higher elongation value in the tensile test was recorded for samples before annealing and the difference was 61%, with the DMTA test shown by Kwiatkowski et al. (2002) [17].

**Figure 12:**
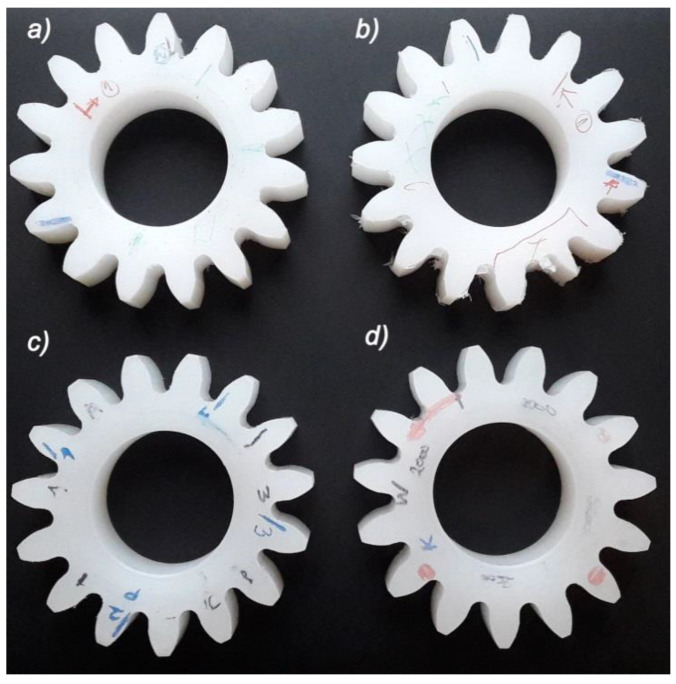
Gear wheels after machining, (**a**) PE1000, (**b**) PE1000 after heat treatment, (**c**) PVDA, (**d**) PVDA after heat treatment.

As a result of the machining of the tested materials, 4 gears were obtained with identical geometrical parameters (Figure 12). In the first stage of testing, all the gears were subjected to a detailed geometric analysis on the coordinated measuring machine, using the professional ZEISS GEAR PRO analysis software. Deviations of the involute outline, pitch and tooth line allowed classification of wheels in the 9th grade of accuracy.

**Figure 13:**
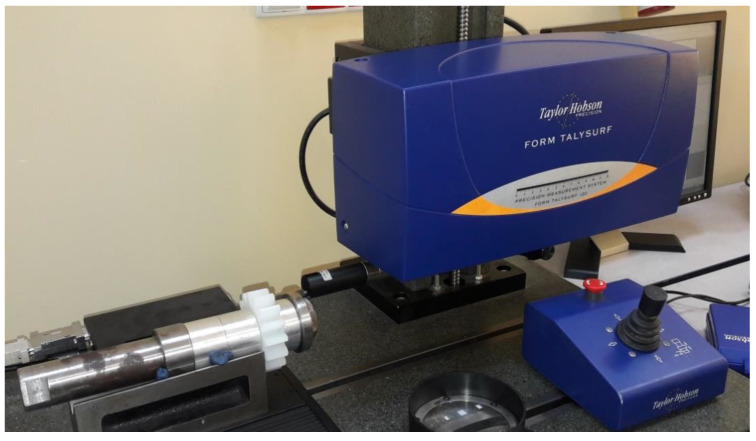
Surface roughness measurement along the longitudinal direction to the machining direction. Taylor Hobson contact profilometer, Talysurf 120.

In the next stage of the research, machined gears were subjected to detailed analysis using the laboratory profile profilograph, Taylor Hobson, Talysurf 120 (Figure 13). Stereoscopy of the surface, roughness of machined tooth in the direction of the profile in the middle of the width of the rim and along the tooth line at the height of the division diameter were measured.

**Figure 14:**
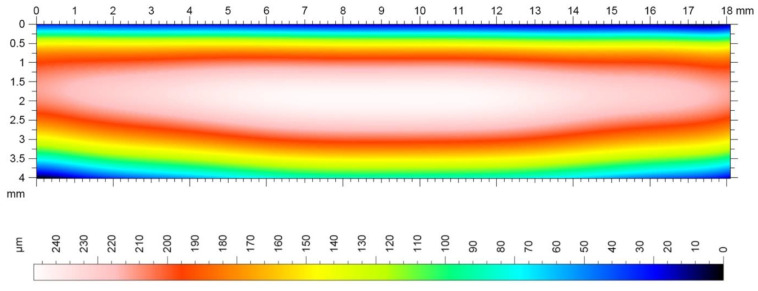
Topography of the machined surface of a gear with a longitudinal modification of the outline, m = 4.5 mm, e = 0.16 mm.

The treated area was characterized by a topography determined to be favorable due to the wheel’s operational properties (spreadability, load-bearing capacity). With the assumed number of tool longitudinal passes of 115 lines, a high machining accuracy (tooth outline, tooth contour) was obtained at the height of the tooth profile as described Sadílek et al. (2015) [18]. The wheel had a barrel correction, which can be seen in the picture, where the central zone significantly dominates the results from side zones, which indicates a “barrel” on the tooth line (Figure 14).

**Figure 15:**
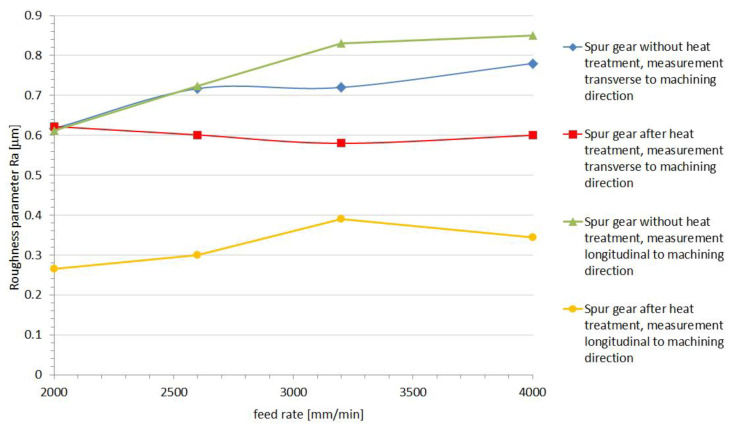
Results of the PVDA roughness measurement.

**Figure 16:**
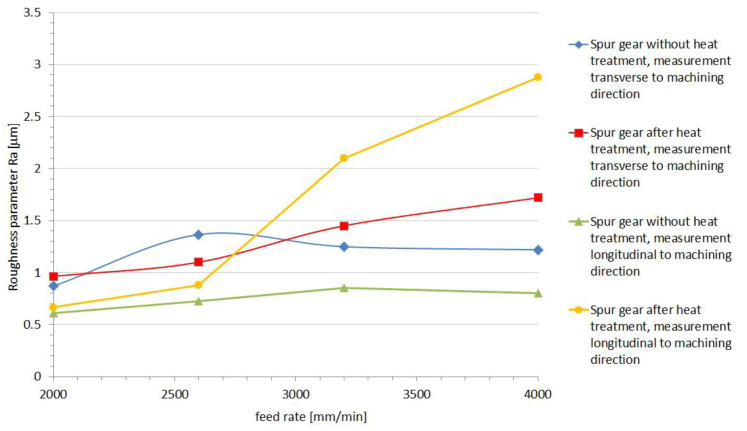
Results of the PE1000 roughness measurement.

Determination of microgeometry of the surface layer is currently the most commonly used measurable feature of surface layer properties. Thanks to the use of Taylor Hobson measurement equipment and the TalyMap Platinium software it was possible with very high accuracy, on the basis of the observed 3D profile, to determine the surface roughness parameters and present them in the form of a graphical interpretation (Figure 15 and Figure 16). The analysis of the machining process carried out allowed us to state that the correct directional structure of the machined surface was formed. The cutting tool at the moment of the machining process contacts the workpiece with only a small part of it on the spherical part, and depending on the height of the tooth’s side, this area was displaced, which could have a significant impact on the surface after machining, as presented by Sadilek et al. (2015) [17]. Changes in surface topography, measured parallel to the machining direction, reflected the effect of plastic modification on the quality of the surface after machining to the greatest extent (Figure 15 and Figure 16). The thermally modified material was much less susceptible to adverse changes in surface roughness. In the case of measurements perpendicular to the machining directions, one could observe a certain identity for both PVDA and PE1000. The roughness characteristics as a function of changes in the feed speed relative to each other did not show significant variation. This conclusion seems to be obvious, if we emphasize that the division of machining allowance was constant for all tool passes. During the machining process, heat is generated at the point of contact of the tool with the workpiece material, and the influence of heat on the chip forming process is a particularly important role in the cutting of polymer materials. After heat treatment, PVDA showed much less susceptibility to fraying at the edges of machined surfaces, formed chips showed greater brittleness and the surface after machining was characterized by lower roughness, with an increased feed rate parameter. For PE1000, the terminal feed rate was recorded much earlier, after which an unsteady increase in roughness occurred. At high feeds, the finished surface became jagged—the chip was not completely separated from the work surface and zones with different roughness could be observed on the treated surface.

**Figure 17:**
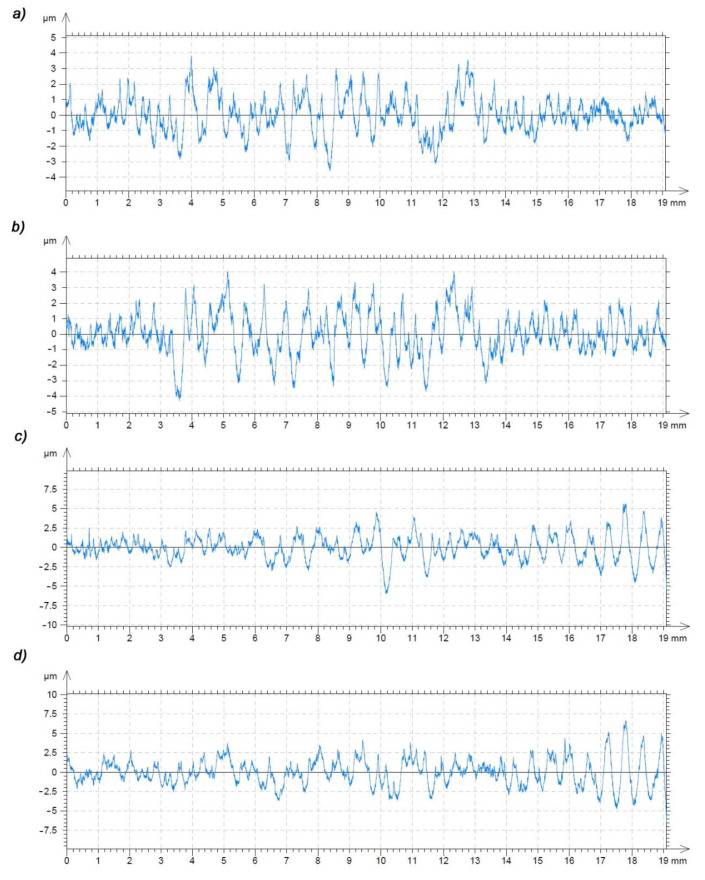
Profile of the roughness parameter R_a_ measured along the tooth line for the size of the feed parameters: (**a**) 2000 (mm/min), (**b**) 2600 (mm/min), (**c**) 3200 (mm/min), (**d**) 4000 (mm/min); PVDA after heat treatment.

**Figure 18:**
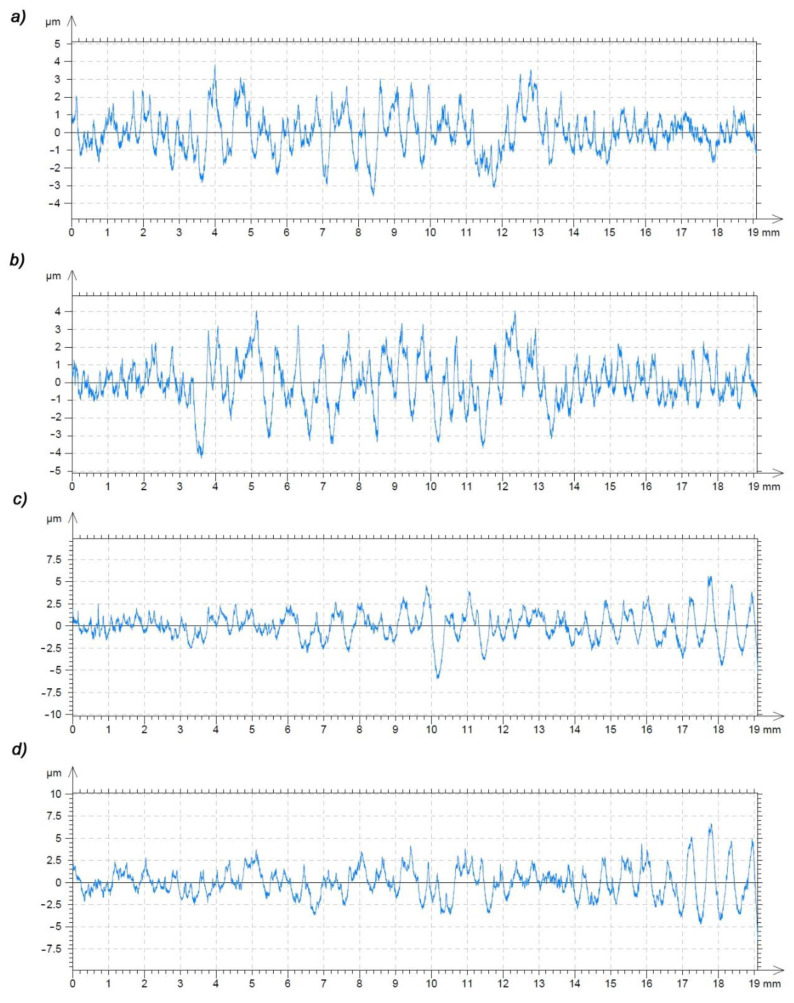
Profile of the roughness parameter R_a_ measured along the tooth line for the size of the feed parameters: (**a**) 2000 (mm/min), (**b**) 2600 (mm/min), (**c**) 3200 (mm/min), (**d**) 4000 (mm/min); PVDA without heat treatment.

Figure 17 and Figure 18 show the roughness profile of the surface of the machined gear tooth measured parallel to the machining direction. The measurement was carried out on a Taylor Hobson laboratory contact profilometer, Talysurf 120 equipped with a diamond measuring needle with a radius of 2 μm. Analyzing the roughness profile, we can see (Figure 18c) significant discrepancies in the surface characteristics of the material after heat treatment (Figure 17) and the native material. The surface of the gear wheel made of PVDA after heat treatment is much less fluctuating in its surface profile. The chip formation process is more predictable and while increasing the feed, there is no more than a normative increase in the surface roughness parameters of the workpiece. It is also noticeable that the edge of the gear wheel is not frayed (Figure 12a,b) as was the case of PE1000, also heat treated. When determining the roughness profile, the parameter R_mr_(c) was also analyzed for the material profile share of the profile, the so-called Abbott-Firestone curve, determined by all tested samples. On the basis of the obtained results, it can be clearly stated that the material share curve for heat-treated PVDA samples is significantly higher and fluctuates in the range of 11–35%. In the case of PE1000, this result in the most advantageous case did not exceed 3%. On this basis, it should be stated that the surface with a full material contribution is definitely more durable—it is a fuller material and its load capacity, and consequently its resistance to surface pressure, are also increasing.

## 4. Summary and Conclusions

From material tests, it can be concluded that PE1000 shows slight changes in thermal properties after annealing. Analyzing the dynamic properties determined by the DMTA method for PE1000, it can be stated that after annealing, the stiffness and damping ability of this material is not improved. The high degrees of crystallinity and high crystallization capacity of polyethylene caused during the annealing of the material did not change too much, which had an influence on the obtained properties of the material being annealed. Tensile strength tests show that PE1000 annealed has similar strength as unheated material and is more flexible. In the case of PVDA Tecaflon tests, it was found that after heating the material it showed a higher melting enthalpy value and the value of the degree of crystallinity increased. When analyzing DMTA thermograms for PVDA Tecaflon, it can be noticed that after annealing, the material is characterized by higher stiffness and better vibration damping. Tensile tests indicated that after annealing, Tecaflon breaks out at a higher strength and with less elongation, which indicates a greater degree of crystallinity and brittleness of the material.

Summing up the conducted research in the field of machining gears, it was found that construction polymers, including the tested PVDA, are characterized by much better susceptibility to scaling with respect to the PA1000 tested as well. Annealing of PVDA improved its strength properties and the brittleness increase improved the material’s ability to delaminate during machining. The machining could be carried out with a significantly higher feed rate as the feed increase did not cause deterioration of the machined surface (roughness (Ra parameter) and material share index (parameter R_mr_ (c))). The structural elements of machines and devices, including gears made of polymer materials, have good mechanical and operational properties, and are increasingly used in the construction of machines. Machining of polymer materials is more demanding in relation to metal processing due to the high thermal susceptibility of material during machining. The conducted research has shown that the machining technology of machine parts and mechanisms including gear wheels made of polymeric materials present large potential for optimization of parameters and machining strategies, and developing guidelines for the selection of cutting tool geometry.

## Figures and Tables

**Table 1:** The temperature range of the tested materials.

SAMPLE	DMTA	DSC
PVDA Tecaflon	−150 °C–180 °C	−50 °C–200 °C
PVDA Tecaflon annealed	−150 °C–180 °C	−50 °C–200 °C
PE1000	−130 °C–140 °C	−50 °C–160 °C
PE1000 annealed	−130 °C–140 °C	−50 °C–160 °C

**Table 2:** The results of material testing using the DSC (differential scanning calorimetry) method.

Sample	Enthalpy of Melting	The Melting Range of Crystalline Phase	Maximum Melting Temperature
PE1000	55.87 J/g	169.3–179.7 °C	175.7 °C
PE1000 annealed	58.33 J/g	163.9–177.3 °C	172.2 °C
PVDA Tecaflon	129.3 J/g	123.2–148.0 °C	141.2 °C
PVDA Tecaflon annealed	146.4 J/g	125.7–145.9 °C	139.8 °C

**Table 3:** Tensile strength test results.

Sample	R_m_ [MPa]	F_max_ [N]	Elongation [%]
PE1000	60.67	2426.8	174
PE1000 annealed	71.86	2874.68	235
PVDA Tecaflon	27.40	1096.1	18.5
PVDA Tecaflon annealed	29.02	1160.64	14.5
